# Incidence of intraepithelial fallopian tube neoplasias in mexican women over 40 years of age that underwent elective hysterectomy

**DOI:** 10.1186/s13048-019-0515-3

**Published:** 2019-06-10

**Authors:** Antonio Gabriel Briseño Campos, Antonio Cruz Rodríguez, Martha Olivia García Perales, Francisco Javier Serna Vela, Diana Gabriela Camarillo Elizalde, María del Consuelo Robles Martínez

**Affiliations:** 1 Gynecology and Obstetrics, Aguascalientes Women’s Hospital, Aguascalientes, Mexico; 2 Gynecological Oncology Department, Aguascalientes Women’s Hospital, Siglo XXI # 109; Morelos, 20298 Aguascalientes, Mexico; 3 Pathology and Cytopathology Laboratory, Aguascalientes, Mexico; 4 Institute of Health Services of the State of Aguascalientes, Aguascalientes, Mexico; 5 Public Health Department of the Health Science Center at the Autonomous University of Aguascalientes, Aguascalientes, Mexico; 6 Gynecology and Obstetrics Department, Aguascalientes Women’s Hospital, Aguascalientes, Mexico

**Keywords:** Malignant; ovarian tumor, Hysterectomy, Salpinge, Prophylactic salpingectomy

## Abstract

**Aims:**

The incidence of intraepithelial neoplasia in the fallopian tubes of women over 40 years of age who had undergone elective hysterectomy was assessed at the Aguascalientes Women’s Hospital.

**Methods:**

An observational, prospective, descriptive study was carried out at the Aguascalientes Women’s Hospital on female patients over 40 years of age who underwent elective hysterectomy between July and October 2017. In these 4 months, 85 patients underwent elective hysterectomy.

**Results:**

In this study, 85 patients who received a hysterectomy for non-oncological reasons were analyzed. Salpinx alterations compatible with intraepithelial neoplasia in the Fallopian tubes were found in 2.4% of the patients studied.

**Conclusions:**

The incidence of intraepithelial neoplasia in the fallopian tubes of high-risk patients at the Aguascalientes Women’s Hospital is 2.4%. Prophylactic salpingectomy is a simple procedure and has the potential to decrease the risk of high-grade ovarian cancer. In premenopausal patients, total abdominal hysterectomy with bilateral salpingectomy should be the procedure most often performed.

## Background

Serous carcinomas are the most common histological subtype of ovarian epithelial cancers and in recent years, a very close relationship has been found between peritoneal and fallopian tube carcinoma based on their histological similarities and clinical behavior. As a result, it has been proposed that these carcinomas (ovarian, peritoneal and fallopian tube) all develop from the fallopian tubes [1]. Indeed, studies of the pathogenesis and heterogeneity of epithelial ovarian cancer concluded that most ovarian cancers originate from the secretory cells of the fallopian tube, although what triggers this neoplastic transformation remains unclear [2–6]. Indeed, the precursors of high-grade serous cancer were identified in the fallopian tubes, which is now defined as Serous Tubal Intraepithelial Cancer (STIC) [7]. This evidence that high-grade serous carcinomas actually arise from the fallopian tube suggests that the incidence of fallopian tube carcinoma may be higher than previously thought.

In this study we set out to study the incidence of STICs in a population of women older than 40 years of age that underwent elective hysterectomy in a single center in Mexico. The results emphasize the benefits to be obtained by performing prophylactic salpingectomy in this population of women in order to reduce the risk of carcinoma.

Ovarian cancer is the second most common gynecological cancer and it is the leading cause of death from gynecological cancer in the United States [8]. Fallopian tube carcinoma represents 0.2% of the cancers in women in the USA [9], while in Mexico, the National Cancer Institute registers more than 2500 new cases of ovarian cancer each year, ranking third in the number of gynecological cancers in our country. Most cases are diagnosed in women between 40 and 59 years of age [10], and while around 95% of malignant ovarian tumors are of epithelial origin,the rest originate from other types of ovarian cells, germ or stromal cells.

There are 2 hypotheses regarding the development of epithelial ovarian cancer:

- Incessant ovulation: trauma in the epithelium of the ovary provokes transformation into malignant cells and indeed, epithelial ovarian cancer is less common in women who undergo periodic suppression of ovulation (contraceptives, pregnancy, lactation) [11].
- Exposure to gonadotropins: persistent exposure of the ovary to gonadotropins and elevated estradiol levels may have a carcinogenic effect [12].

In women under 50 years of age, the incidence of ovarian cancer increases by 2% each year, while its incidence increases by 11% after 50 years of age [13]. The women at highest risk are those within the period of fertility, those who experience menarche before the age of 12 or menopause after the age of 52 [14], the use of hormone replacement therapy [15], nulliparity [16], endometriosis (which increases the risk for clear cell cancer, low grade endometrioid cancer and cancer of the serous type) [17], polycystic ovary [18], and the use of copper IUDs [19]. Smoking increases the risk of mucinous ovarian cancer and asbestos exposure augments the risk of epithelial ovariancancer [13]. The mutation of some genes has also been associated with an increased risk of epithelial ovarian cancer, such as BRCA1 and BRCA2, BRIP1, RAD51C and RAD51D [14, 15]. Conversely, women who use oral contraceptives or levonorgestrel IUDs and women with multiparity have a lower risk of developing epithelial ovarian cancer [14, 20–22]. In fact, term pregnancies reduce the risk of epithelial ovarian cancer by 8% for each pregnancy [23] and multiple pregnancies [24], as well as pregnancies under 35 years of age, are also protective factors [18, 25]. Ovulation inducers do not increase the risk of this type of tumor [26].

In 1990, risk reduction surgery for epithelial ovarian cancer began to be implemented in patients with mutations in BRCA1 and BRCA2. At this time hidden neoplasms began to be identified in the fallopian tubes, even in association with microscopic invasion in high-grade serous carcinomas and high-grade intraepithelial neoplasia, suggesting that the fallopian tube could be the origin of high-grade non-uterine pelvic serous carcinomas. Bilateral salpingectomy-oophorectomy reduces mortality in patients carrying mutations inBRCA1 and BRCA2, individuals at a high risk of developing high-grade serous carcinomas. Moreover, bilateral salpingectomy is recommended in patients with no risk factors for epithelial ovarian cancer when a definitive method of family planning is required and in patients undergoing hysterectomy, although the ovaries are preserved in these procedures. The consequences of salpingectomy have been analyzed in all women and in high-risk groups, focusing on morbidity, ovarian function and clinical application [27]. Opportunistic salpingectomy is performed to reduce the risk of epithelial ovarian cancer, yet some of these women may develop peritoneal carcinoma. Unilateral salpingectomy-oophorectomy, hysterectomy without oophorectomy and tubal ligation all reduce the risk of epithelial ovarian cancer, more so when considering non-serous carcinomas and in individuals under 35 years of age) [28–30].

## Materials and methods

An observational, descriptive, prospective study was carried out over a period of 4 months. Patients older than 40 years of age with a preoperative diagnosis of a benign pathology and that were recommended a surgical protocol for abdominal hysterectomy were included in this study. Patients under 40 years of age and not recommended a full surgical protocol were excluded from the study and a patient that refused to participate in the study was eliminated from the cohort.

The dependent variable assessed was STIC development and the independent variables were age, parity, menarche, pregnancy, null parity, family history of ovarian cancer, smoking and endometriosis. The type of sample is non-probabilistic and the sample size was the total of the 85 patients included on the study.

The phatologic samples were stained with hematoxylin/eosin; these were prepared in paraffin with 2 mm sections.

The study was carried out in accordance with the guidelines for General Health Law regarding research and specifically, with that regulating descriptive studies carried out on humans. The study also complies with the rights of patients and respects their rights to confidentiality by adhering to the Helsinki and Belmont declarations regarding human research.

## Results

**Fig. 1:**
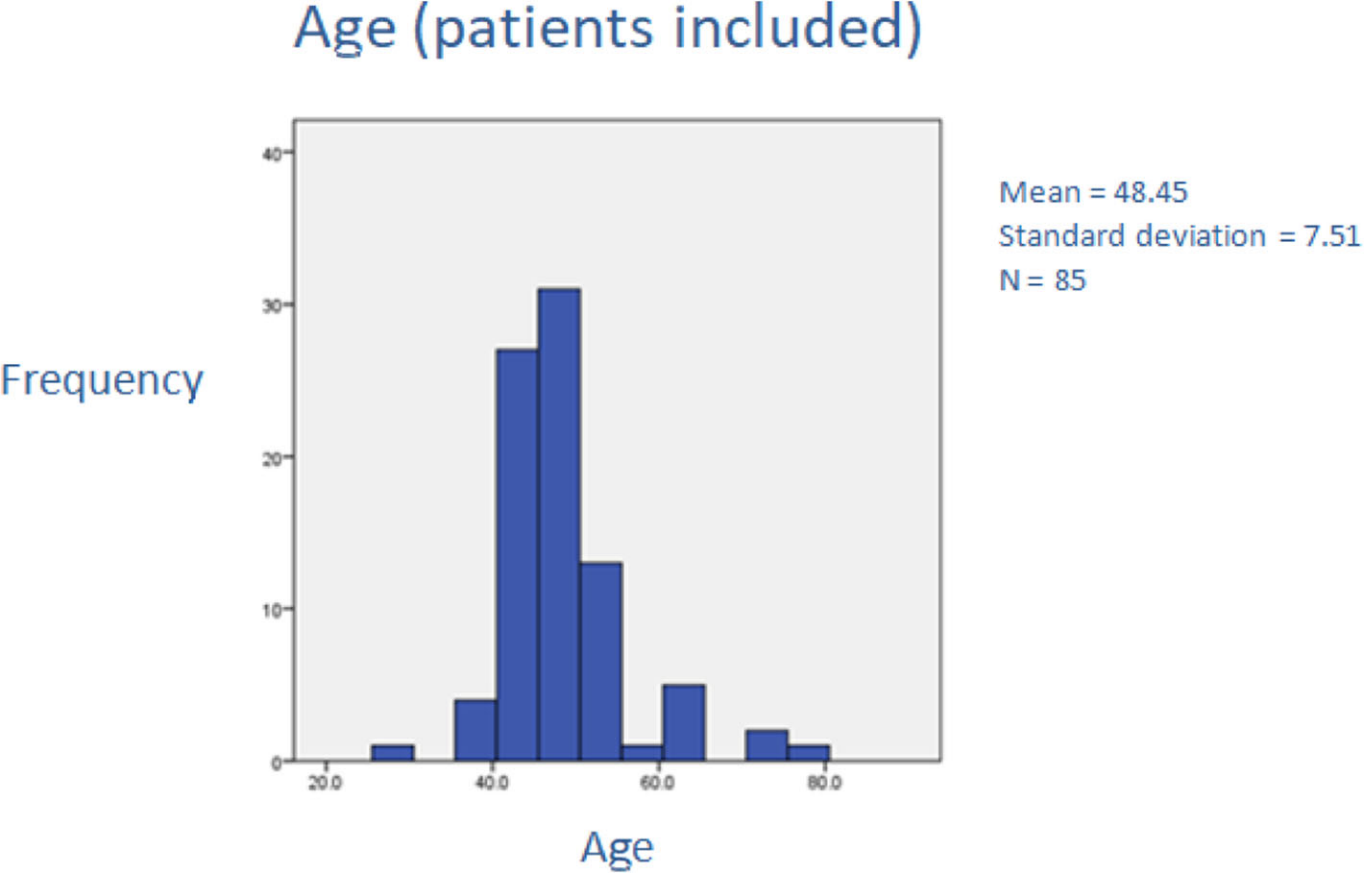
The age of the patients that underwent hysterectomy and opportunistic salpingectomy (range 40–76 years of age)

The mean age of the cohort was 48.45 years (± 7.51: Fig. 1).

Gynecological and obstetric history are shown in Table 1, but they show no relevant data.

**Table 1:** Gynecological and Obstetric Background of the patients undergoing opportunistic salpingectomy

Gynecological -Obstetric History Of The Patients Studied							
		Menarche	Gestations	Births	Ectopic Pregnancies	Abortions	Cesareans
No.	Valid	85	85	85	85	85	85
	Lost	0	0	0	0	0	0
Mean		11.7	3.7	2.7	0.012	0.4	0.62
Median		12.0	3.0	2.0	0	0	0
Mode		11.0	3.0	2.0	0	0	0
Standard Deviation		1.5	2.6	2.5	0.1	0.7	1.0
Variance		2.2	6.9	6.6	0.01	0.5	1.1
Rank		6.0	13.0	13.0	1.0	4.0	4.0
Minimum		9.0	0	0	0	0	0
Maximum		15.0	13.0	13.0	1.0	4.0	4.0

The main indication for undergoing hysterectomy among the subjects included in the study was uterine myomatosis, with a frequency of 54 cases (63.5%), consistent with the existing literature (Table 2), then pelvic organ prolapse was the surgery indication (9.4%). Postmenopausal bleeding, uterine bleeding refractory to treatment and mature cyst teratoma showed low frequency (1.2%).

**Table 2:** Pre-surgical diagnosis

Hysterectomy Indications				
	Frequency	Percentage	Valid percentage	Accumulated percentage
Adenomyosis	7	8.2	8.2	8.2
Endometrial hyperplasia	4	4.7	4.7	12.9
Uterine myomatosis	54	63.5	63.5	76.5
Endometrial polyps	3	3.5	3.5	80.0
Pelvic organ prolapse	8	9.4	9.4	89.4
Postmenopausal bleeding	1	1.2	1.2	90.6
Uterine bleeding refractory to treatment	1	1.2	1.2	91.8
Mature cyst teratoma	1	1.2	1.2	92.9
Anexial tumor	5	6.0	6.0	100.0
TOTAL	85	100.0	100.0	

Total hysterectomy with bilateral salpingo-oophorectomy was the procedure performed most often, in 50.7% of the patients including abdominal and vaginal surgery (Table 3^a^). The age group of patients studied corresponds to women at perimenopause and menopause. It is striking that right oophorectomy was performed more frequently.

**Table 3:** Surgical procedure performed in the population undergoing opportunistic salpingectomy

Surgical Procedure Performed				
	Frequency	Percentage	Valid percentage	Accumulated percentage
Abdominal subtotal hysterectomy + bilateral salpingectomy + right oophorectomy	1	1.2	1.2	1.2
Abdominal total hysterectomy + bilateral salpingectomy	13	15.3	15.3	16.5
Abdominal total hysterectomy + bilateral salpingectomy + right oophorectomy	17	20.0	20.0	36.5
Abdominal total hysterectomy + bilateral salpingectomy + left oophorectomy	6	7.1	7.1	43.6
Abdominal total hysterectomy + bilateral salpingo-oophorectomy	37	43.6^a^	43.6	87.2
Abdominal total hysterectomy + right salpingo-oophorectomy	3	3.5	3.5	90.7
Vaginal hysterectomy + bilateral salpingectomy + left oophorectomy	1	1.2	1.2	92.9
Vaginal hysterectomy + bilateral salpingo-oophorectomy	6	7.1^a^	7.1	100.0
Total	85	100.0	100.0	

**Fig. 2:**
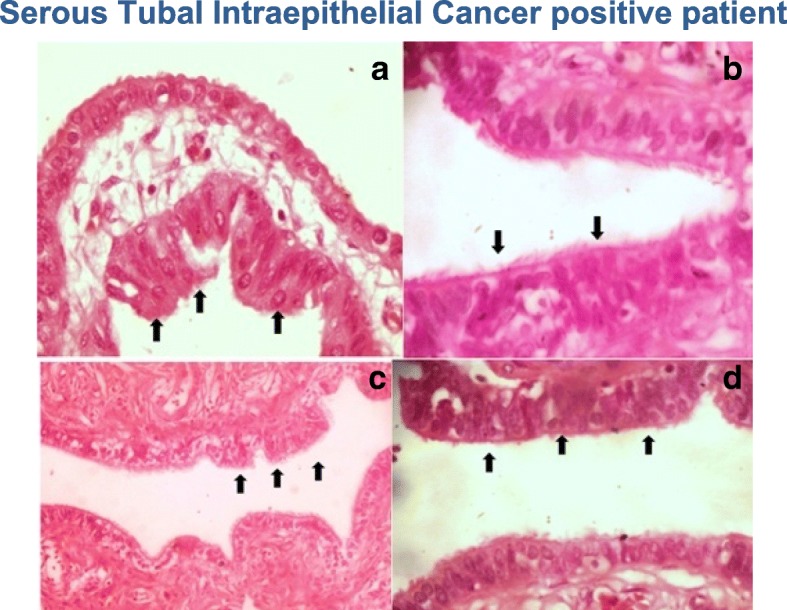
**a**-**b**.STIC positive patient S-17644. **c**-**d**.STIC positive patient S-17754. The black arrows indicate nuclear enlargement, loss of polarity and atypia

We failed to find any type of pathological alterations in 81.2% of the patients, whereas the incidence of patients who presented with serous carcinoma of the ovary was 4.8% and the preoperative diagnosis was that of an adnexal tumor that was subjected to further study. One case of a perivascular tumor of epithelioid cells in the salpinge was detected, while two patients had STIC, corresponding to 2.4% of the patients who entered the study (Figs. 2). The patients in which STIC was detected were 44 and 55 years of age, both with a pre-surgical diagnosis of uterine myomatosis. The patients with STIC had a history of a family member with breast cancer, whereas none of the patients had a family history of ovarian cancer.

## Discussion

Since 1990, the rise of risk-reduction surgery in patients carrying mutations in BRCA led to the detection of hidden neoplasms in the Fallopian tubes of patients over 40 years of age. Indeed, high-grade epithelial ovarian cancer is associated with precursor lesions in the fallopian tube epithelium [3–7]. Here, we assessed the incidence of STICs in a population of Mexican women of at least 40 years of age that underwent elective hysterectomy at our clinical center. The average age of the patients studied here was 48 years, similar to those in a study conducted in Japan in 2016 [6], and the individuals in which STIC was detected here were 44 and 55 years old. However, we do not know the incidence of STIC in patients under 40 years of age, largely because it is less common to perform a hysterectomy in patients of that age.

The main indication for performing a hysterectomy in our study was uterine myomatosis, which is consistent with the existing literature. The STIC positive patients experienced menarche at 11 and 10 years of age, and while one of the patients had 2 pregnancies during her reproductive life (one delivery and one abortion), the other patient had a single cesarean section. Endometriosis was not found in patients positive for STIC and a BRCA test was not carried out due to the lack of financial resources. However, it is notable that the incidence of STIC in the BRCA positive population is 30–40% in the literature [6].

Total hysterectomy with bilateral salpingo-oophorectomy was the procedure most often performed in this cohort (50.7% of cases), with bilateral salpingectomy also carried out frequently (44.8%of cases). The age group of the individuals studied corresponds to a perimenopausal and menopausal population, and accordingly, the STIC-positive patients underwent total abdominal hysterectomy with bilateral salpingectomy. In low risk patients there is no preventive measure to reduce the risk of ovarian cancer, yet it would appear that prophylactic salpingectomies could reduce the risk of developing high-grade ovarian cancer [3, 27]. Nevertheless, the incidence of STIC in low-risk women is an issue that has been little studied and while an incidence of STIC of 3.2% was reported in low risk women in Japan in 2016 [6], no such data is available in Mexico. From our data it appears that we could reduce the risk of high-grade epithelial ovarian cancer in 2 out of every 100 patients that undergo opportunist salpingectomy. If we also take into account the study conducted previously in Japan in 2016 [6], a total of 208 patients have been studied of whom 6 were diagnosed with STIC, an incidence of 2.88%.

In our institution 1814 bilateral tubal occlusions are performed in the population of childbearing age. The lack of applicability as a multicenter study to increase the sample is considered a limitation of the study. This represents an opportunity to perform opportunistic salpingectomy and thereby reduce the risk of high-grade ovarian cancer in 240 patients per 100,000.

## Conclusion

The incidence of STIC in a low risk population in Mexico is 2.4%. Prophylactic salpingectomy should be performed in all patients undergoing hysterectomy as it reduces the risk of developing high-grade ovarian cancer. The main indication for hysterectomy in our institution was uterine myomatosis, and the procedure most commonly performed in perimenopausal and menopausal patients was total abdominal hysterectomy with bilateral salpingo-oophorectomy. This should also be the most common procedure offered to premenopausal patients.

## Perspectives

In this study, we set out to determine the incidence of STIC in a population of low risk patients. However, it would be prudent to analyze alterations toTP53 in such low risk populations in order to study alterations to the fallopian tubes. We believe that this study may be useful to improve the gynecological attention given to women and to promote the use of prophylactic salpingectomy in women of this age, with the aim of reducing the occurrence of high grade ovarian cancer in this population.
